# Differential Expression Patterns of Lynx Proteins and Involvement of Lynx1 in Prepulse Inhibition

**DOI:** 10.3389/fnbeh.2021.703748

**Published:** 2021-11-03

**Authors:** Yasmine Sherafat, Edison Chen, Valeria Lallai, Malia Bautista, James P. Fowler, Yen-Chu Chen, Julie Miwa, Christie D. Fowler

**Affiliations:** ^1^Department of Neurobiology and Behavior, University of California, Irvine, Irvine, CA, United States; ^2^Department of Biological Sciences, Lehigh University, Bethlehem, PA, United States

**Keywords:** nicotinic acetylcholin receptor (nAChR), cholinergic signaling, sensory gating, learning and memory, cognitive flexibility

## Abstract

Negative allosteric modulators, such as lynx1 and lynx2, directly interact with nicotinic acetylcholine receptors (nAChRs). The nAChRs are integral to cholinergic signaling in the brain and have been shown to mediate different aspects of cognitive function. Given the interaction between lynx proteins and these receptors, we examined whether these endogenous negative allosteric modulators are involved in cognitive behaviors associated with cholinergic function. We found both cell-specific and overlapping expression patterns of lynx1 and lynx2 mRNA in brain regions associated with cognition, learning, memory, and sensorimotor processing, including the prefrontal cortex (PFC), cingulate cortex, septum, hippocampus, amygdala, striatum, and pontine nuclei. Since lynx proteins are thought to play a role in conditioned associations and given the expression patterns across brain regions, we first assessed whether lynx knockout mice would differ in a cognitive flexibility task. We found no deficits in reversal learning in either the lynx1^–/–^ or lynx2^–/–^ knockout mice. Thereafter, sensorimotor gating was examined with the prepulse inhibition (PPI) assessment. Interestingly, we found that both male and female lynx1^–/–^ mice exhibited a deficit in the PPI behavioral response. Given the comparable expression of lynx2 in regions involved in sensorimotor gating, we then examined whether removal of the lynx2 protein would lead to similar behavioral effects. Unexpectedly, we found that while male lynx2^–/–^ mice exhibited a decrease in the baseline startle response, no differences were found in sensorimotor gating for either male or female lynx2^–/–^ mice. Taken together, these studies provide insight into the expression patterns of lynx1 and lynx2 across multiple brain regions and illustrate the modulatory effects of the lynx1 protein in sensorimotor gating.

## Introduction

The cholinergic system controls a variety of complex cognitive processes, such as attention, sensorimotor gating, cognitive flexibility, reinforcement, learning, and memory (Court et al., 1999; Miwa et al., 2006, 2011; Tekinay et al., 2009; Zhang et al., 2010; de la Salle et al., 2013; Freitas et al., 2013; Chen et al., 2018, 2020; Solari and Hangya, 2018; Anderson et al., 2020; Sherafat et al., 2021). Integral to the cholinergic system are the nicotinic acetylcholine receptors (nAChRs). Binding of the endogenous agonist acetylcholine induces a conformational change that opens the channel, allowing for the influx of Na^+^ and Ca^2+^ and the efflux of K^+^, followed by a desensitized state (Lipsius, 1982; Fuentealba et al., 2004). The nAChRs exhibit various allosteric binding sites, which allow for modulation of the pharmacokinetics associated with receptor activation and desensitization (Le Novere et al., 2002). The lynx1 and lynx2 proteins are classified as negative allosteric modulators of the cholinergic system through their actions in reducing the activity of the nAChRs in the presence of an agonist (Ibanez-Tallon et al., 2002; Kobayashi et al., 2014; Nichols et al., 2014; George et al., 2017). Therefore, lynx proteins dampen the cholinergic system’s activity, which has been proposed to subsequently underlie changes in memory, learning, and plasticity (Miwa et al., 2006; Morishita et al., 2010).

At the cell membrane, the lynx proteins are mainly thought to associate with nAChRs while anchored to the membrane through a GPI link (George et al., 2017), but depending on the isoform, lynx proteins may also be released into the extracellular space to exert actions on the membrane surface (Dessaud et al., 2006; Holford et al., 2009; Tekinay et al., 2009; Bychkov et al., 2019; Dong et al., 2020). In addition to their actions on membrane-bound nAChRs, lynx proteins may also affect the trafficking of nAChR subunits to the membrane via association in the endoplasmic reticulum (Nichols et al., 2014; Miwa et al., 2019). The ability of lynx proteins to modulate these cholinergic processes through both extracellular and intracellular mechanisms has important implications for cognitive aspects of neurological and psychiatric diseases (Smith et al., 2018; Artoni et al., 2020; Miwa, 2020). Indeed, prior studies in mice lacking the lynx1 (lynx1^–/–^) and lynx2 (lynx2^–/–^) proteins have provided foundational insights into the function of these endogenous modulators. For instance, removal of lynx1 augments cholinergic processing, leading to enhanced associative learning in a fear conditioning task and expanded critical period for visual plasticity (Miwa et al., 2006; Morishita et al., 2010; Bukhari et al., 2015; Sajo et al., 2016). Removal of lynx2 has been shown to increase anxiety associated behavior and decrease social interaction (Dessaud et al., 2006; Miwa et al., 2012). While the actions of the lynx proteins are considered to be inhibitory to excitatory signaling, it is important to note that the cell-type specific pattern of expression may lead to opposing effects. For example, expression on GABAergic cells leads to net inhibition of the inhibitory neurons, thereby increasing gain modulation (Disney et al., 2007; Morishita et al., 2010). However, the extent of modulation imposed by the lynx proteins leading to downstream behavioral effects has not yet been fully elucidated.

Prepulse inhibition (PPI) is an operational measure of sensorimotor gating that is disrupted in many neurological and psychiatric disorders, including Schizophrenia, Huntington’s Disease, Alzheimer’s Disease, and dementia (Swerdlow et al., 1995; Bakshi and Geyer, 1998; Takeuchi et al., 2011; Kucinski et al., 2012). Sensorimotor gating is a feature of the nervous system in adjusting a response to consequent stimuli based on prior experiences (Golubic et al., 2019). The modulation of the neural response induced by stimuli is a cognitive ability that is considered to be essential to maintaining function in everyday life (Golubic et al., 2019). Given that the PPI protocol can be implemented in both rodent and human models, this assessment is characterized as having translational relevance (Swerdlow et al., 1999), and interestingly, the septum, pedunculopontine tegmental nucleus (PPTg), pontine reticular nucleus (PnC), nucleus accumbens (NAcc), and amygdala have been shown to regulate PPI-relevant sensorimotor and cognitive processing across species (Swerdlow and Geyer, 1993; Swerdlow et al., 1995, 2007; Wan and Swerdlow, 1997; Bakshi and Geyer, 1998; Schell et al., 2000; Baldan Ramsey et al., 2011; Takeuchi et al., 2011; Kucinski et al., 2012; Kiziltan et al., 2018; Jin et al., 2019; Sullivan et al., 2019; Cano et al., 2021). For instance, optogenetic activation of the cholinergic neurons in the PPTg has been shown to increase prepulse facilitation (Azzopardi et al., 2018). Given that studies have implicated multiple brain regions in PPI, this highlights the dynamic complexity of brain circuit signaling to mediate such a response.

Reversal learning is another cognitive process that refers to the ability to quickly adjust behavior in the face of changing situations (Izquierdo et al., 2017). Studies have found that brain regions such as the medial prefrontal cortex (PFC), striatum, hippocampus, and cingulate cortex play an important role in reversal learning (Castane et al., 2010; Dalton et al., 2016; Vila-Ballo et al., 2017; Stolyarova et al., 2019). For example, chemogenetic inhibition of the cingulate cortex affects confidence enhanced reversal learning (Stolyarova et al., 2019). Similar to PPI, reversal learning is disrupted in a variety of neurological and psychiatric disorders, including substance abuse, obsessive compulsive disorder, Parkinson’s disease, and Schizophrenia (Dargis et al., 2017; Tezcan et al., 2017; Bechard et al., 2018; Kleinmans and Bilkey, 2018; Levy-Gigi et al., 2019). In a reversal learning paradigm, subjects are trained to discriminate between stimuli, one of which is rewarded when chosen. After the discrimination has been learned, the outcomes associated with the two stimuli are reversed and subjects are tested on their ability to appropriately adjust their behavior (Izquierdo et al., 2017). This classical reversal learning paradigm is also translationally relevant, as it has been used in both humans and rodent models (Schoenbaum et al., 2000; Fellows and Farah, 2003).

In these studies, we first examined whether lynx1 and lynx2 were expressed in brain regions implicated in cognitive function. Based on our findings, we then focused our studies on mouse models with targeted deletion of the *Lynx1* gene or the *Lynx2* gene via homologous recombination (Miwa et al., 2006; Tekinay et al., 2009). Since lynx1 has been shown to be involved in cue-associated conditioning, we first assessed male and female lynx1 knockout (lynx1^–/–^) and wildtype (lynx1^+/+^) mice in a cued reversal learning task. Next, we performed this reversal assessment in the male and female lynx2^–/–^ and lynx2^+/+^ mice. Thereafter, we examined whether the male and female lynx1^–/–^ and lynx1^+/+^ mice differ in their baseline startle response and sensorimotor gating for PPI. Finally, given the similar expression of lynx2 in regions mediating sensorimotor gating, we also examined male and female lynx2^–/–^ and lynx2^+/+^ mice in the PPI assessment. Together, our data suggest differential roles of lynx1 and lynx2 proteins in startle reactivity and sensorimotor gating responses in males and females.

## Materials and Methods

### Mice

Male and female adult mice on a C57BL/6J background with null mutations in the *Lynx1* or *Lynx2* gene and their respective wildtype littermates were bred in our animal facilities. Mice were 8–15 weeks of age, group housed and maintained in a humidity- and temperature- controlled (22°C) vivarium on a reverse 12 h: 12 h light: dark cycle. Prior to all behavioral assessments, mice were habituated in the room with the experimenter across 2 days, and behavioral tests were conducted during the active, dark phase of the light cycle. All procedures were conducted in strict accordance with the NIH Guide for the Care and Use of Laboratory Animals and approved by the University of California Irvine IACUC.

### Lynx1 and Lynx2 Genotyping

At 21 days of age, pups were weaned, and tails were clipped for genetic analysis. Lynx1 littermates were genotyped with the following primers: Lynx1 WT Forward (CTGGAGTGCCACGTGTGTGCC), Lynx1 KO Forward (GCCAGCTTGGCGTGAAGTTCC), and Lynx1 WT/KO Reverse (CGTTTGAGTGGATCTGGCTTGGGG). The band for the lynx1 wildtype allele was detected at 470 bp and for the lynx1 knockout allele at 200 bp. Lynx2 littermates were genotyped with: Lynx 2 WT/KO Forward (CCACCGAATCTCCCAAATCC), Lynx2 KO Reverse (CCCTGGCAATTAACCCTAA), Lynx2 WT Reverse (TCCTCCACTACTCCCCTTTCTGAC). The band for the lynx2 wildtype allele was detected at 200 bp and for the lynx2 knockout allele at 400 bp.

### RNAScope

Brain tissue was examined to determine *Lynx1* and *Lynx2* mRNA expression in the PFC, cingulate cortex, septum, NAcc, striatum, amygdala, hippocampus, PPTg, and PnC. Male and female adult C57BL/6J wildtype (*n* = 5 per sex) were anesthetized with ketamine-xylazine and perfused through the ascending aorta with 0.9% saline followed by 4% paraformaldehyde in 0.1 M PBS, pH 7.4. Thereafter, brains were removed and postfixed for 2 h in paraformaldehyde, followed by cryoprotection in 30% sucrose for ∼72 h. Brain sections were cut on a cryostat at 35-μm intervals. Three sections per brain region were analyzed for each subject. Afterward, brain tissue was processed for RNAscope Multiplex Fluorescent assay (Advanced Cell Diagnostics) as previously described (Sherafat et al., 2020). Briefly, sections were placed in an incubator for 30 min at 60°C then treated at 100°C for 6 min in target retrieval solution (Advanced Cell Diagnostics). Sections were dehydrated in 100% ethanol and treated with protease (Advanced Cell Diagnostics, catalog #322380). RNA hybridization probes included *Lynx1* (Advanced Cell Diagnostics, catalog # 449078) and *Lynx2* (Advanced Cell Diagnostics, catalog #447088-C2), which were labeled with Opal 520 and Opal 570 (PerkinElmer), respectively. Slides were then counterstained and cover slipped with Vectashield containing DAPI (Vector Laboratories) and imaged with a Leica fluorescence microscope.

### Reversal Learning

Mice were assessed for reversal learning as previously described (Pushkin et al., 2019; Figure 4A). Subjects were mildly food restricted (85–90% of their free feeding weight) and trained to press a lever in an operant chamber for 20 mg food pellets (5TUM, Test Diet) under a fixed ratio 5, time-out 20 s (FR5TO20s) schedule of reinforcement. Each session was performed using 2 retractable levers (1 active, 1 inactive) for a 1 h session. Completion of the response criteria on the active lever resulted in the delivery of a food pellet, and activation of a cue light above the lever for the 20 s time-out duration. Responses on the inactive lever were recorded but had no scheduled consequences. Once stable responding was achieved (criteria > 30 pellets per session across 3 daily consecutive sessions), the lever assignment was reversed in the subsequent session to examine cognitive flexibility on the reversal day. Specifically, the previous inactive lever became active, for which food pellets were delivered with the FR5TO20s schedule of reinforcement. In contrast, the previously active lever now became inactive, in which responses were recorded without scheduled consequence. Therefore, the mice were food trained for ≥ 5 days to achieve stable responding, and then they were tested in the reversal session the following day. Thus, the baseline session represents the day immediately prior to reversal, whereas the reversal session represents the day of reversal. All behavioral responses were automatically recorded by MedAssociates software.

### Acoustic Startle Testing

Startle and PPI testing were examined in startle chambers (SR-LAB, San Diego Instruments, United States), using an experimental design as previously described (van Enkhuizen et al., 2015; Figure 6A). Briefly, mice were placed into the startle chambers, which consisted of a Plexiglas cylinder, 5 cm in diameter, resting on a platform in a ventilated sound-attenuating box. Speakers mounted 33 cm above the cylinders produced the acoustic stimuli, and movements of the animal were transduced by piezoelectric accelerometers mounted under the cylinders and stored by the computer interface. The rodent’s startle response data in the SR-Lab System is directly recorded from an accelerometer that produces as an analog output voltage signal in the millivolt (mV) range. The rodent startle response includes movement of all 4 limbs crouching dynamically. Thus, the force of the movements generate voltage which the accelerometer records. Immediately prior to the PPI session, mice were permitted a 5 min acclimation period at 60 dB in the chamber. Thereafter, the PPI test consisted of a startle block comprising five 120 dB startle pulses, and the mean of these responses represents the baseline startle response value. Thereafter, five prepulse blocks were presented, which consisted of a 120 dB startle pulse preceded by either a 70 dB prepulse, 75 dB prepulse, 80 dB prepulse, or 85 dB prepulse. Each block presented the specific prepulses in a randomized manner. Prepulses were 20 ms in duration, the interstimulus intervals were 50 ms, and the startle pulses were 40 ms. In the programmed protocol, the intertrial interval had a random variable duration of 4–12 s. To calculate percent PPI, the following equation was used: Percent PPI = [(S – P)/S] × 100, where S is the average baseline startle response per subject (mV) and P is the average response following each prepulse and startle pairing (mV). Thus, a lower value of percent PPI indicates a greater response in the presence of a prepulse, thereby demonstrating a decrease in sensorimotor gating; conversely, a higher percent PPI is indicative of more efficient sensorimotor gating.

### Statistical Analysis

Data were analyzed using GraphPad Prism software (La Jolla, CA). For comparisons of two groups, data were analyzed using unpaired, two-tailed *t*-tests. If greater than two groups, data were analyzed using repeated measures (RM) two-way analysis of variance (ANOVA) with a Geisser-Greenhouse correction. The Geisser-Greenhouse correction was applied since we had no *“a priori”* assumptions for equal variability of differences within each group condition. This allows for matched values to be stacked into a subcolumn and individual variances to be computed for each comparison, in accordance with the repeated measures two-way ANOVA. Given variability in the baseline startle for PPI, we performed a ROUT outlier test, and the following were identified as outliers and removed from analysis: one male Lynx1^–/–^, one female Lynx1^–/–^ and one female Lynx1^+/+^. For multiple comparisons, the Sidak’s *post hoc* test was employed, with statistical correction for multiple comparisons. The criterion for significance was set at α = 0.05.

## Results

### Lynx1 and Lynx2 mRNA Expression in the Brain

**FIGURE 1:**
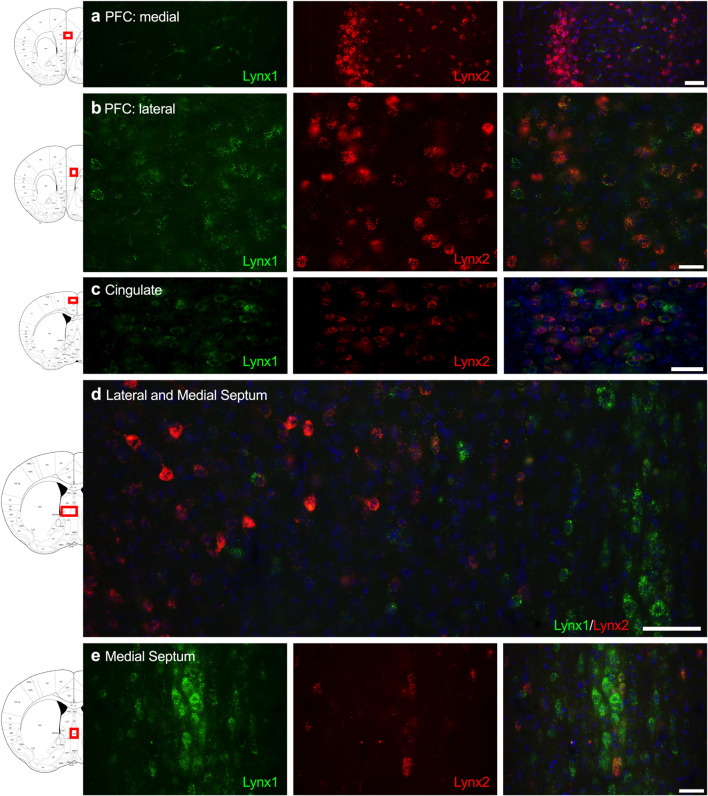
Expression of lynx1 and lynx2 mRNA in the prefrontal cortex (PFC), cingulate cortex, and septum. **(a)** Lynx2 (red), but not lynx1 (green), is highly expressed in cells of the medial PFC. **(b)** In the lateral PFC, a different pattern emerged with cells expressing either lynx1 or lynx2, or both lynx1 and lynx2, in this region. **(c)** In the cingulate cortex, most cells expressed both lynx1 and lynx2, although cells were still identified that only expressed lynx1 or lynx2. **(d)** Lower magnification of the septum shows distinct region-specific differential expression of lynx1 and lynx2 in the medial and lateral regions, respectively. **(e)** Higher magnification of the medial septum shows a high density of lynx1-positive cells. The red boxes on the brain plates to the left of each row illustrate the region shown in the respective microscopy images. Blue: DAPI. Scale bar for (**a–c,e)** 40 μm. Scale bar for **(d)** 100 μm.

**FIGURE 2:**
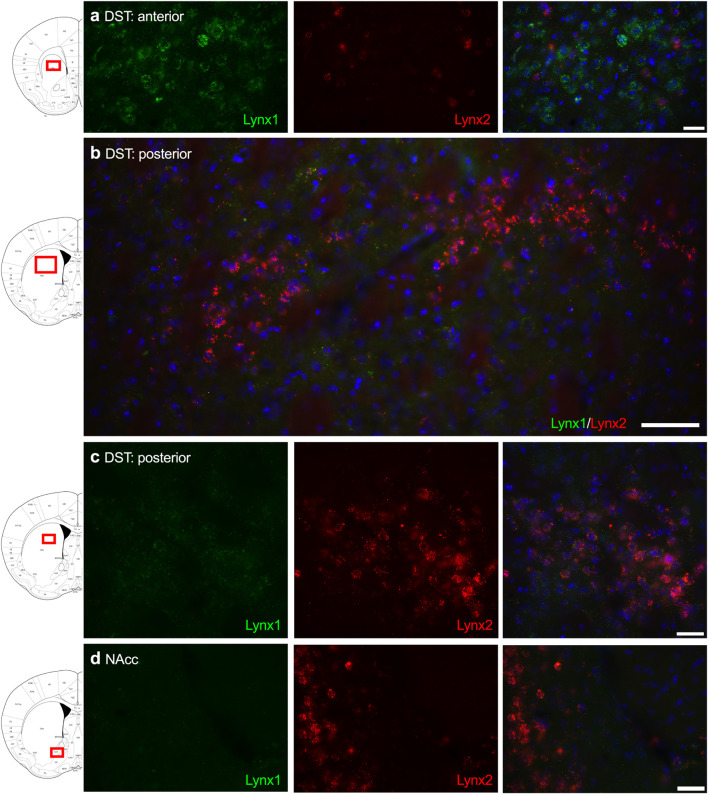
Expression of lynx1 and lynx2 mRNA in the striatum. **(a)** High levels of lynx1 (green) were found in anterior dorsal striatum (DST), with lower levels of lynx2 (red). **(b)** Lower magnification of the posterior DST region shows clustered cells expressing lynx2. **(c)** Higher magnification of the posterior DST further illustrates dense expression of lynx2 in a cluster region. **(d)** In the nucleus accumbens (NAcc), preferential expression of lynx2 was observed. The red boxes on the brain plates to the left of each row illustrate the region shown in the respective microscopy images. Blue: DAPI. Scale bar for **(a,c,d)** 40 μm. Scale bar for **(b)** 100 μm.

**FIGURE 3:**
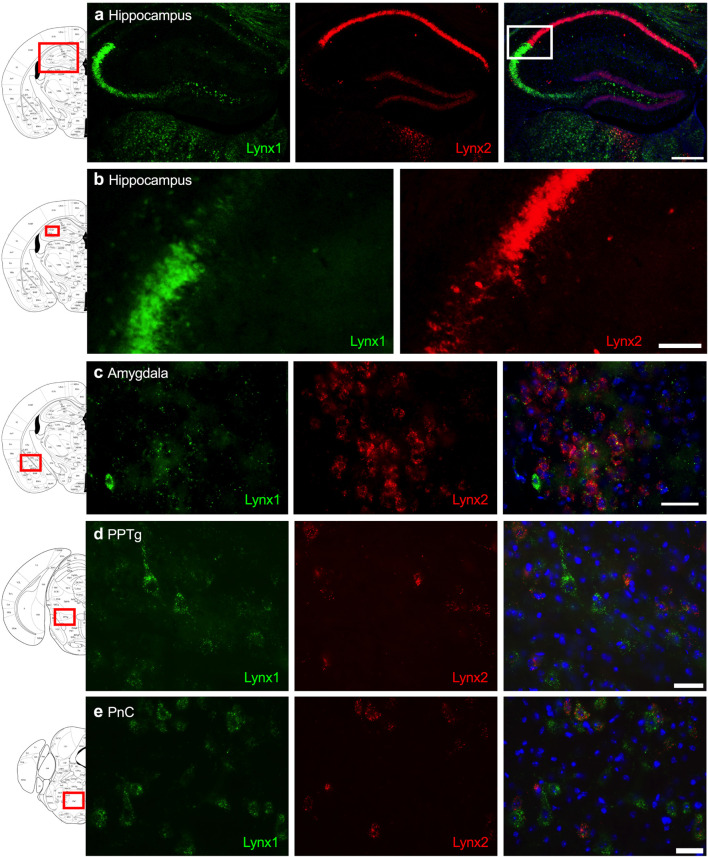
Expression of lynx1 and lynx2 mRNA in the hippocampus, amygdala, pedunculopontine tegmental nucleus (PPTg), pontine reticular nucleus (PnC). **(a)** Distinct patterns of lynx1 and lynx2 expression were found in the dorsal hippocampus. Interestingly, lynx1 mRNA (green) was densely expressed in the CA2 and CA3 region, whereas lynx2 mRNA (red) was highly expressed in the CA1 region. White box denotes the higher magnification image shown in **(b)**. **(b)** Higher magnification of the hippocampus shows distinct differences in the density of lynx1 and lynx2 expression across the CA regions. **(c–e)** In the central amygdala **(c)**, PPTg **(d)** and PnC **(e)**, cells were found to express lynx1, lynx2, or co-expression both isoforms. The red boxes on the brain plates to the left of each row illustrate the region shown in the respective microscopy images. Blue: DAPI. Scale bar for **(a)** 100 μm. Scale bar for **(b–e)** 40 μm.

Given that the PFC, cingulate cortex, and septum have been implicated in cognitive function, we examined lynx1 and lynx2 mRNA expression across these brain regions. We found selective expression of lynx2 in the more medial region of the PFC (Figure 1a), whereas the lateral PFC exhibited high lynx1 and lynx2 co-expression patterns (Figure 1b), with some cells exhibiting selective expression of either lynx1 or lynx2. A similar pattern was found in the cingulate cortex (Figure 1c), in which single- and co-expression patterns were evidenced. Interestingly in the septum, a distinct pattern emerged, in which lynx2 appeared to be preferentially expressed in the lateral septum (Figure 1d), whereas the medial septum exhibited dense expression of lynx1 (Figure 1e); however, these expression patterns were not exclusive as few cells expressed lynx1 in the lateral septum and lynx2 in the medial septum. Next, we examined striatal regions and observed interesting differentiation depending on the subregion. Specifically, in the more anterior regions of the DST (Figure 2a), lynx1 was predominantly expressed, but in the posterior DST, higher levels of lynx2 were present (Figure 2c). Of further interest, when examined at lower magnification (Figure 2b), the localization of lynx2 in the dorsal striatum resembled striosome expression patterns (Brimblecombe and Cragg, 2017), although this localization will need to be confirmed in further studies. In the ventral striatum, we also found a higher density of expression for lynx2 in the NAcc (Figure 2d). In the dorsal hippocampus (Figure 3a), we found a high density of lynx1 and lynx2 with cell-layer selective expression patterns. Interestingly, when examined at higher magnification, distinct differences were found between CA3/CA2 and CA1, with preferential expression of lynx1 or lynx2, respectively (Figure 3b). We also found both lynx1 and lynx2 expression in the central amygdala (Figure 3c), PPTg (Figure 3d), and PnC (Figure 3e), in which both co-localization and independent expression of either lynx1 or lynx2 were observed. Finally, we did not evidence any distinct differences between males and females in the overall expression patterns of lynx1 or lynx2; however, this will need to be examined more precisely with other techniques in future studies.

### Involvement of Lynx1 in Reversal Learning

**FIGURE 4:**
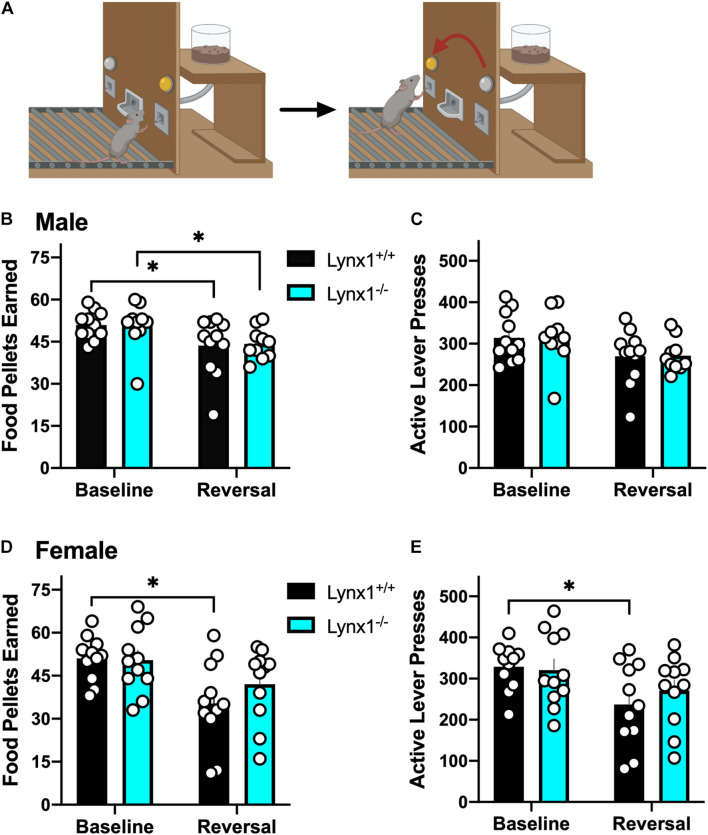
Assessment of the role of lynx1 in cognitive flexibility. **(A)** Schematic demonstrating the reversal learning paradigm, in which the active lever is reversed after the mice have established a baseline level of responding for food reward. **(B,C)** Male lynx1^–/–^ and lynx1^+/+^ mice (*n* = 10–11/group) displayed a significant decrease in the number of food pellets earned **(B)**, but no difference in the number of active lever presses **(C)**, comparing the baseline to the reversal session. **(D,E)** Female lynx1^–/–^ and lynx1^+/+^ mice (*n* = 11/group) were also assessed in the reversal learning task. The female lynx1^–/–^ mice exhibited a similar level of lever pressing behavior across sessions, with no differences in the number of food pellets earned **(D)** or active lever presses **(E)** in the reversal session compared to their baseline level of responding. In contrast, the lynx1^+/+^ mice displayed a significant decrease in both of these measures for the reversal session. ^∗^*p* < 0.05. Individual data points shown as white circles, and the bars represent mean ± SEM. Image created with BioRender.com.

Since lynx1 was expressed in brain regions involved in behavioral learning (Kosaki and Watanabe, 2012; Kawai et al., 2015), we first examined the effects of *Lynx1* gene knockout in a reversal learning operant food training task (Figure 4A). We found that both male lynx1^–/–^ and lynx1^+/+^ mice exhibited a significant decrease in the number of food pellets earned during the reversal session, as compared to the baseline session (Figure 4B) [RM two-way ANOVA: Genotype: *F*_(__1_, _19__)_ = 0.0067, *p* = 0.9356; Session: *F*_(__1_, _19__)_ = 14.46, *p* = 0.0012; Interaction: *F*_(__1_, _19__)_ = 0.0340, *p* = 0.8557]. The *post hoc* test comparing session revealed a significant decrease in the number of food rewards earned in both the male lynx1^–/–^ (*p* = 0.0430) and lynx1^+/+^ (*p* = 0.0187) mice, when comparing the baseline vs. reversal session. However, the decrease was not evident in the number of lever presses on the active lever, as both the male lynx1^–/–^ and lynx1^+/+^ mice exhibited high levels of lever pressing in both sessions (Figure 4C) [RM two-way ANOVA: Genotype: *F*_(__1_, _19__)_ = 0.0018, *p* = 0.9669; Session: *F*_(__1_, _19__)_ = 9.523, *p* = 0.006; Interaction: *F*_(__1_, _19__)_ = 0.0012, *p* = 0.9727; *post hoc*, lynx1^–/–^ (*p* = 0.0947) and lynx1^+/+^ (*p* = 0.0701)]. For the females, the lynx1^–/–^ mice demonstrated no significant decrease in food pellets earned during reversal compared to baseline, whereas their lynx1^+/+^ littermates displayed a statistically significant decrease (Figure 4D) [RM two-way ANOVA: Genotype: *F*_(__1_, _20__)_ = 0.6945, *p* = 0.4145; Session: *F*_(__1_, _20__)_ = 11.61, *p* = 0.0028; Interaction: *F*_(__1_, _20__)_ = 1.050, *p* = 0.3177]. *Post hoc* tests revealed significant differences for female lynx1^+/+^ (*p* = 0.0104), but not the lynx1^–/–^ (*p* = 0.2036), mice in the number of rewards earned on baseline vs. reversal day. When comparing active lever presses between sessions, similar effects were found with the female lynx1^–/–^ mice (Figure 4E) [RM two-way ANOVA: Genotype: *F*_(__1_, _20__)_ = 0.2623, *p* = 0.6142; Session: *F*_(__1_, _20__)_ = 7.248, *p* = 0.014; Interaction: *F*_(__1_, _20__)_ = 0.6276, *p* = 0.4375]. The *post hoc* test revealed a significant decrease for female lynx1^+/+^ (*p* = 0.0453), but not lynx1^–/–^ (*p* = 0.3506), mice in the number of active lever presses earned between the baseline and the reversal session. Therefore, the female lynx1^–/–^ mice were highly efficient in the lever reversal task by adapting to the modified requirements. Together, these data indicate that removal of lynx1 does not induce deficits in cognitive flexibility.

### Involvement of Lynx2 in Reversal Learning

**FIGURE 5:**
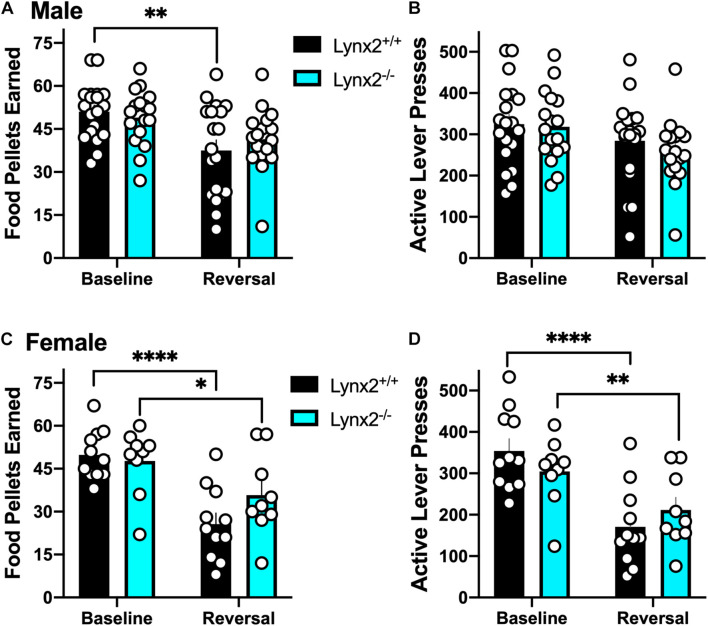
Assessment of the role of lynx2 in cognitive flexibility. **(A,B)** Male lynx2^–/–^ and lynx2^+/+^ mice (*n* = 17–19/group) were examined in the reversal learning task. The lynx2^+/+^ mice displayed a significant decrease in the number of food pellets earned **(A)**, but no difference in the number of active lever presses **(B)**, comparing the reversal session to the baseline day. **(C,D)** Both lynx2^+/+^ and lynx2^–/–^ female mice (*n* = 9–11/group) exhibited a significant decrease in number of food pellets earned **(C)** and active lever presses **(D)** in the reversal session compared to their baseline level of responding. ^∗^*p* < 0.05, ^∗∗^*p* < 0.01, ^****^p < 0.0001. Individual data points shown as white circles, and the bars represent mean ± SEM.

We next examined lynx2 mice in the reversal learning task. We found that male lynx2^+/+^, but not lynx2^–/–^, mice exhibited higher variability in the number of food pellets earned during the reversal session, as compared to the baseline session (Figure 5A) [RM two-way ANOVA: Genotype: *F*_(__1_, _34__)_ = 0.1102, *p* = 0.7420; Session: *F*_(__1_, _34__)_ = 12.96, *p* = 0.001; Interaction: *F*_(__1_, _34__)_ = 1.065, *p* = 0.3094]. *Post hoc* tests revealed a significant decrease in rewards earned for male lynx2^+/+^ (*p* = 0.0038), but not the lynx2^–/–^ (*p* = 0.1647), mice on the baseline vs. reversal day. However, for active lever presses, neither male lynx2^–/–^ nor lynx2^+/+^ mice exhibited any differences between sessions with high rates of responding in both sessions (Figure 5B) [RM two-way ANOVA: Genotype: *F*_(__1_, _34__)_ = 0.4322, *p* = 0.5154; Session: *F*_(__1_, _34__)_ = 6.878, *p* = 0.0130; Interaction: *F*_(__1_, _34__)_ = 0.2682, *p* = 0.6079; *post hoc*, lynx2^–/–^ (*p* = 0.0741) and lynx2^+/+^ (*p* = 0.2516)]. For the females, both lynx2 genotypes demonstrated a significant decrease in food pellets earned during reversal compared to the baseline session (Figure 5C) [RM two-way ANOVA: Genotype: *F*_(__1_, _18__)_ = 0.7632, *p* = 0.3938; Session: *F*_(__1_, _18__)_ = 40.96, *p* < 0.0001; Interaction: *F*_(__1_, _18__)_ = 4.757, *p* = 0.0427]. *Post hoc* tests revealed significant differences for female lynx2^–/–^ mice (*p* = 0.0214) and lynx2^+/+^ (*p* < 0.0001) in the number of rewards earned on baseline vs. reversal day. Similarly, when comparing active lever presses between sessions, both female lynx2^–/–^ and lynx2^+/+^ displayed a significant decrease on the reversal session (Figure 5D) [RM two-way ANOVA: Genotype: *F*_(__1_, _18__)_ = 0.0132, *p* = 0.9097; Session: *F*_(__1_, _18__)_ = 55.10, *p* < 0.0001; Interaction: *F*_(__1_,_18__)_ = 5.934, *p* = 0.0255]. *Post hoc* tests revealed significant decrease for female lynx2^–/–^ mice (*p* = 0.0069) and lynx2^+/+^ (*p* < 0.0001) in the number of active lever presses on the baseline vs. reversal session. Together, these findings indicate that lynx2 does not significantly modulate processes underlying cognitive flexibility in the reversal task.

### Involvement of Lynx1 in Sensorimotor Gating With Prepulse Inhibition

**FIGURE 6:**
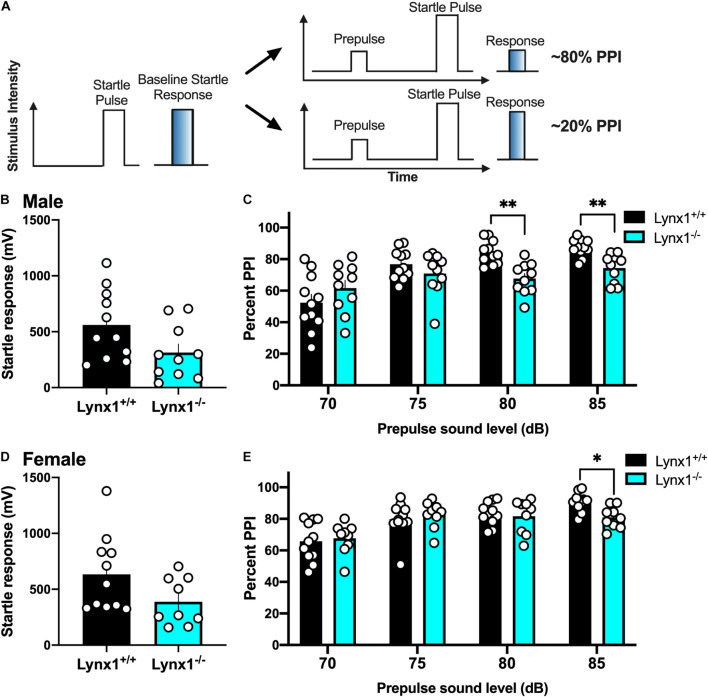
Involvement of lynx1 in sensorimotor gating. **(A)** Schematic demonstrating the prepulse inhibition (PPI) procedure, in which a lower percent PPI is indicative of decreased sensorimotor gating following the prepulse paired with a startle pulse. Analysis of the baseline startle response (e.g., as represented by the left in **A**) was first performed, followed by the prepulse and startle pulse paired sequence, with the prepulse of varying decibels (dB). **(B)** Male lynx1^+/+^ and lynx1^–/–^ mice (*n* = 10–11/group) exhibited no differences in their baseline startle response. **(C)** Following the presence of a prepulse, male lynx1^–/–^ mice exhibited a decrease in their percent PPI at the 80 and 85 dB prepulse compared to the lynx1^+/+^ mice. **(D)** Female lynx1^–/–^ and lynx1^+/+^ mice (*n* = 9–11/group) exhibited no genotype differences in baseline startle response. **(E)** Following the presence of a prepulse, female lynx1^–/–^ mice exhibited a significant decrease in percent PPI at the 85 dB prepulse compared to the lynx1^+/+^ mice. ^∗^*p* < 0.05, ^∗∗^*p* < 0.01. Individual data points shown as white circles, and the bars represent mean ± SEM. Image created with BioRender.com.

To examine the role of lynx1 in sensorimotor gating, we conducted the PPI assessment in the male and female lynx mice (Figure 6A). First, male lynx1^–/–^ mice exhibited no significant differences in their baseline startle response compared to lynx1^+/+^ littermates (Figure 6B) [two-tailed *t*-test: *t*_(__19__)_ = 2.013, *p* = 0.0585]. However, in the PPI assessment, significant differences between the responses of the lynx1^–/–^ and lynx1^+/+^ mice were exhibited (Figure 6C) [RM two-way ANOVA: Genotype: *F*_(__1_, _19__)_ = 2.713, *p* = 0.1160; dB: *F*_(__2_._053_, _39_._00__)_ = 30.37, *p* < 0.0001; Interaction: *F*_(__3_, _57__)_ = 9.268, *p* < 0.0001]. Specifically, the *post hoc* test revealed a significant decrease in percent PPI for the lynx1^–/–^ mice at the 80 dB (*p* = 0.0023) and 85 dB (*p* = 0.0067), but not 70 dB or 75 dB, compared to lynx1^+/+^ levels. Female lynx1^–/–^ mice also exhibited no differences in baseline startle response compared to lynx1^+/+^ littermates (Figure 6D) [two-tailed *t*-test: *t*_(__18__)_ = 1.875, *p* = 0.0771], but differences were found in the percent PPI between genotypes (Figure 6E) [RM two-way ANOVA: Genotype: *F*_(__1_, _18__)_ = 0.3020, *p* = 0.5894; dB: *F*_(__2_._589_, _46_._61__)_ = 27.17, *p* < 0.0001; Interaction: *F*_(__3_, _54__)_ = 3.215, *p* = 0.0299]. The *post hoc* test indicated a decrease in the percent PPI for the lynx1^–/–^ mice at the 85 dB (*p* = 0.0129), but not at the 70, 75, or 80 dB levels, compared to lynx1^+/+^ mice. Thus, removal of lynx1 led to a deficit in the percent PPI in both males and females, suggesting that the presence of lynx1 is an important modulator of sensorimotor gating.

### Involvement of Lynx2 in Sensorimotor Gating With Prepulse Inhibition

**FIGURE 7:**
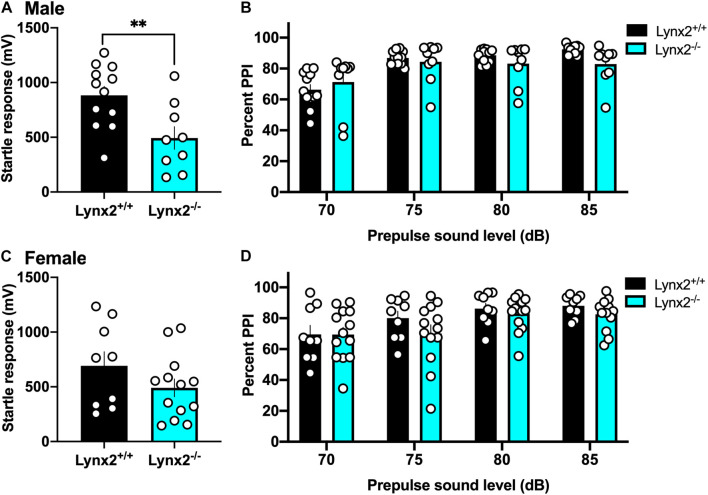
Lack of association between lynx2 and sensorimotor gating. **(A)** For the baseline startle response, male lynx2^–/–^ and lynx2^+/+^ mice (*n* = 9–12/group) were examine, and the lynx2^–/–^ exhibited a decrease in their startle reactivity compared to lynx2^+/+^ littermates. **(B)** Following the presence of a prepulse, male lynx2^–/–^ mice exhibited no differences in their percent PPI compared to the lynx2^+/+^ mice. **(C,D)** Female lynx2^–/–^ and lynx2^+/+^ mice (*n* = 9–13/group) exhibited no differences in the baseline startle response **(C)** or percent PPI **(D)**. ^∗∗^*p* < 0.01. Individual data points shown as white circles, and the bars represent mean ± SEM.

Next, we examined the involvement of the lynx2 isoform in sensorimotor gating to determine if these effects were specific to the interactions of lynx1 with cholinergic signaling. We found that male lynx2^–/–^ mice exhibited a decreased baseline startle response (Figure 7A) [two-tailed *t*-test: *t*_(__19__)_ = 2.984, *p* = 0.0076], which was not found in females (Figure 7C) [two-tailed *t*-test: *t*_(__20__)_ = 1.390, *p* = 0.1799]. However, in contrast to the findings with male lynx1^–/–^ mice, the male lynx2^–/–^ mice exhibited no statistical differences in their percent PPI responses (Figure 7B) [RM two-way ANOVA: Genotype: *F*_(__1_, _19__)_ = 0.5608, *p* = 0.4631; dB: *F*_(__1_._486_, _28_._23__)_ = 52.19, *p* < 0.0001; Interaction: *F*_(__3_, _57__)_ = 6.166, *p* = 0.0011]. Although a main effect for dB and interaction effects were found, the *post hoc* did not reveal any differences between the genotypes at each dB level [*post hoc*, lynx1^–/–^ vs. lynx2^–/–^ at 70 db (*p* = 0.9325), 75 db (*p* = 0.9732), 80 db (*p* = 0.6979), and 85 db (*p* = 0.1806)]. Female lynx2^–/–^ mice also exhibited no differences in their PPI response compared to lynx2^+/+^ littermates, although a main effect across dB was still present (Figure 7D) [RM two-way ANOVA: Genotype: *F*_(__1_, _20__)_ = 1.051, *p* = 0.3176; dB: *F*_(__2_._057_, _41_._15__)_ = 9.534, *p* = 0.0004; Interaction: *F*_(__3_, _60__)_ = 0.7208, *p* = 0.5435]. These data indicate that although lynx2 may mediate the initial startle response in males, it does not appear to be involved in the sensorimotor gating response.

## Discussion

In these studies, we characterized brain region expression patterns and examined the involvement of the endogenous allosteric modulators, lynx1 and lynx2, in reversal learning, startle reactivity and sensorimotor gating. Interestingly, we found that the absence of the lynx1 and lynx2 proteins did not induce deficits in either males or females with reversal learning. In contrast, a deficit in sensorimotor gating was found in both male and female lynx1^–/–^ mice, as they exhibited decreased PPI behavioral responses at moderate to higher prepulse decibels. Surprisingly, although lynx2 is expressed in similar brain regions as lynx1, removal of the lynx2 protein did not alter the PPI behavioral responses in either male or female mice, indicating differential functional roles of these isoforms.

### Lynx1, Lynx2, and Cognitive Flexibility

In the present studies, we demonstrated that lynx1 and lynx2 are localized throughout many brain regions that have been implicated in cognitive flexibility, including the medial PFC, striatum, hippocampus, and cingulate cortex. Previous studies have not examined lynx2 behaviorally in cognitive functions such as learning and memory, but it has been demonstrated that lynx2 can blunt nicotine-induced upregulation of the α4β2 containing nAChRs, which are involved in learning and memory function (Sadleir et al., 2013; Wu et al., 2015). Prior studies have found that lynx1^–/–^ mice exhibit an enhanced fear conditioned response compared to their wildtype littermates for cued, but not contextual, associated tasks (Miwa et al., 2006). In support of these findings, lynx1^–/–^ mice also exhibit normal contextual memory with passive avoidance conditioning and in the Morris Water maze (Miwa et al., 2006). Given these data, we hypothesized that lynx1^–/–^ mice would exhibit an altered response in the lever reversal task, which involves cue-associated learning with the pairing of a cue light and food reward upon completion of response criteria on the active lever. However, while the female lynx1^–/–^ mice appeared to switch their responses more efficiently to receive food rewards on the previously inactive lever, there was not a significant genotype effect. Since this task involves a visual light cue above the active lever, it’s important to note that lynx1 has been implicated in regulating spine dynamics in the visual cortex (Miwa et al., 2006; Morishita et al., 2010; Bukhari et al., 2015; Sajo et al., 2016). However, these effects were found during critical periods for visual plasticity, and adult lynx1^–/–^ mice demonstrate normal dendritic complexity and spine density in primary visual cortical neurons (Sajo et al., 2016), which supports the current results as no deficits were evidenced. Furthermore, the presence of lynx1 has been suggested to serve as a neuroprotective factor in aged animals, such that older lynx1^–/–^ mice develop loss of nerve fibers in the dorsal striatum, a brain region implicated in habitual behavior and reversal learning (Kobayashi et al., 2014; Bonnavion et al., 2019). However, the neuroprotective role of lynx1 appears to emerge in mice after 13 months of age (Kobayashi et al., 2014), which exceeds the age of the mice in the current study. Thus, it would be of interest in further studies to examine whether deficits in the reversal task would emerge in older lynx1^–/–^ animals and whether the administration of nicotine would lead to differential interactive effects between the lynx isoforms and receptor signaling in cognitive flexibility measures.

### Lynx1, Lynx2, and Sensorimotor Gating

Lynx1 and lynx2 were found to be expressed throughout brain regions, with distinct single- and co-expression patterns. The regional hippocampal expression evidenced is consistent with prior literature, in which lynx1 and lynx2 are differentially expressed in the CA1, CA2, and CA3 regions, as determined previously with *in situ* hybridization (Miwa et al., 1999; Dessaud et al., 2006; Tekinay et al., 2009). The septum, PPTg, PnC, NAcc, hippocampus and amygdala have been shown to regulate PPI-relevant sensorimotor and cognitive processing across species (Swerdlow and Geyer, 1993; Swerdlow et al., 1995, 2007; Wan and Swerdlow, 1997; Bakshi and Geyer, 1998; Schell et al., 2000; Baldan Ramsey et al., 2011; Takeuchi et al., 2011; Kucinski et al., 2012; Azzopardi et al., 2018; Kiziltan et al., 2018; Jin et al., 2019; Sullivan et al., 2019; Cano et al., 2021), thereby supporting the notion that presynaptic or postsynaptic cholinergic modulation via the lynx proteins could underlie such cognitive processing. Indeed, we found that both male and female lynx1^–/–^ mice exhibited decreased PPI behavioral responses following moderate prepulse decibels, an effect which was not found in the lynx2 mice. Importantly, lynx1^–/–^ mice have been shown to exhibit normal auditory thresholds (Takesian et al., 2018), indicating that the current findings cannot be attributed to deficits in auditory processing. Of further interest, removal of lynx1 results in the differential expression of genes potentially associated with Schizophrenia (Smith et al., 2018), suggesting that lynx1 may function upstream to regulate the expression of other signaling molecules involved in psychiatric symptomology. It is also possible that differences in anxiety-related behaviors may confer altered responses in the PPI assessment. However, lynx1^–/–^ mice do not exhibit differences in anxiety-associated behaviors, as previously assessed in the elevated plus maze and open field test (Miwa et al., 2006). Interestingly, lynx2^–/–^ does result in increased anxiety-associated behaviors (Tekinay et al., 2009), although we found no differences in sensorimotor gating with PPI in these mice. Thus, the anxiety-associated phenotype did not contribute to an altered sensorimotor gating effect. However, we did observe a lower baseline startle response with the initial series of auditory pulses in male lynx2^–/–^ mice. These results are intriguing given that (1) differences were not found in the females and (2) deficits in the baseline startle response did not correlate with the subsequent differences between groups in percent PPI. Thus, each of these measures supports the relevance of the findings for lynx1 in mediating behavioral motor responses to sensory stimuli.

### Importance of Examining Both Males and Females

Our studies included both males and females, unlike other prior reports in the field that have focused on males (Miwa et al., 2006). Our goal was to potentially reveal behavioral differences occurring *within* each sex in the absence of lynx1 or lynx2. Of note, estrogen has been found to act as a positive allosteric modulator for nAChRs by increasing the open state probability of the channel with ligand binding (Curtis et al., 2002). Further, estrogen receptors localized on cholinergic terminals in the hippocampus have been shown to increase acetylcholine release (Packard et al., 1996; Towart et al., 2003), and cholinergic modulation of inhibitory neurons in the hippocampus can be modified by estrogen’s effects on synaptogenesis (Murphy et al., 1998; Rudick et al., 2003). In addition to these activational effects of estrogen, organizational effects during development can result in functional differences. For instance, sex differences have been documented in the membrane localization of estrogen receptors and subsequent excitation of hippocampal circuits (Oberlander and Woolley, 2016). Since testosterone can be converted locally into estrogen in the male brain (Schulster et al., 2016), it is possible that this hormone exerts differential actions on cholinergic signaling based on the level of expression and localization of estrogen receptors within brain regions, such as the hippocampus. Taken together, these prior studies demonstrate both organizational and activational effects of estrogen and highlight the notion that estrogen may interact with lynx at different developmental stages to modulate function of the nAChRs. Thus, given that males and females fluctuate in their hormonal levels across varying daily cycles, a future study controlling for the relative levels of testosterone and estrogen will be essential to more clearly define such an interaction.

## Conclusion

These studies demonstrate a role for lynx1 in various aspects of cognitive processing with sex-specific effects. It will be important in future studies to ascertain the cell-type specific patterns of expression for the lynx proteins within different brain circuits and to precisely assess the potential competition of positive and negative allosteric modulators of the nAChRs at the synaptic level between sexes. In addition, given the use of nicotine with tobacco cigarettes and e-cigarettes among the population, it should be noted that nicotine’s actions on the nAChR may also be modulated by the presence of the lynx proteins, such as that previously demonstrated with nicotine-mediated nociception, dexterity and grip strength, and glutamate signaling in animal models (Tekinay et al., 2009; Miwa and Walz, 2012; Nissen et al., 2018). Therefore, it will be of interest in future studies to determine if the administration of nicotine or other nAChR ligands interacts with lynx processing to modulate cognitive flexibility or the sensorimotor gating response. Furthermore, it will be interesting to examine lynx1 and lynx2 knockdown or overexpression in specific brain regions on reversal learning and PPI, either in the presence or absence of nAChR ligands. Finally, these findings also highlight a potential role for therapeutic targeting of lynx1 and/or its allosteric binding site on nAChRs to promote more efficient sensorimotor processing by increasing the activity of lynx1 signaling mechanisms.

## Data Availability Statement

The original contributions presented in the study are included in the article/supplementary material, further inquiries can be directed to the corresponding author/s.

## Ethics Statement

The animal study was reviewed and approved by the University of California Irvine IACUC.

## Author Contributions

YS, VL, and CF were responsible for the study concept and design. YS, EC, VL, MB, JF, and Y-CC contributed to the acquisition of animal data. YS, EC, MB, and CF conducted the data analysis and interpreted the findings. JM provided the lynx1 and lynx2 knockout breeders, genotyping protocol and conceptual guidance. All authors contributed to drafting the manuscript, providing critical revision of the manuscript for important intellectual content, and then approved final version for publication.

## Conflict of Interest

JM was co-founder of Ophidion, Inc., a biotechnology company. The remaining authors declare that the research was conducted in the absence of any commercial or financial relationships that could be construed as a potential conflict of interest.

## Publisher’s Note

All claims expressed in this article are solely those of the authors and do not necessarily represent those of their affiliated organizations, or those of the publisher, the editors and the reviewers. Any product that may be evaluated in this article, or claim that may be made by its manufacturer, is not guaranteed or endorsed by the publisher.
